# Gut Microbiome-Driven Strategies to Overcome Immunotherapy Resistance in Microsatellite-Stable Colorectal Cancer

**DOI:** 10.3390/cancers18162538

**Published:** 2026-08-07

**Authors:** Lidia Boldeanu, Alice Elena Ghenea, Alina Elena Ciobanu Plasiciuc, Mihail Virgil Boldeanu, Rodica Pădureanu, Mohamed-Zakaria Assani, Vlad Pădureanu, Isabela Siloși, Marius Bogdan Novac, Ancuța-Ramona Boicea Camen

**Affiliations:** 1 Department of Microbiology, Faculty of Medicine, University of Medicine and Pharmacy of Craiova, 200349 Craiova, Romania; lidia.boldeanu@umfcv.ro (L.B.); alice.ghenea@umfcv.ro (A.E.G.); 2 Department of Health and Motricity, Faculty of Medical and Behavioral Sciences, “Constantin Brâncuși” University of Târgu-Jiu, 210185 Târgu-Jiu, Romania; 3 Department of Immunology, Faculty of Medicine, University of Medicine and Pharmacy of Craiova, 200349 Craiova, Romania; mohamed.assani@umfcv.ro (M.-Z.A.); isabela.silosi@umfcv.ro (I.S.); 4 Department of Internal Medicine, Faculty of Medicine, University of Medicine and Pharmacy of Craiova, 200349 Craiova, Romania; rodica.padureanu@umfcv.ro (R.P.); vlad.padureanu@umfcv.ro (V.P.); 5 Doctoral School, University of Medicine and Pharmacy of Craiova, 200349 Craiova, Romania; 6 Department of Anesthesiology and Intensive Care, University of Medicine and Pharmacy of Craiova, 200349 Craiova, Romania; marius.novac@umfcv.ro; 7 Department of Occupational Medicine, University of Medicine and Pharmacy of Craiova, 200349 Craiova, Romania; ancuta.boicea@umfcv.ro

**Keywords:** colorectal cancer, microsatellite-stable colorectal cancer, gut microbiome, immune resistance, immune checkpoint inhibitors, *Fusobacterium nucleatum*, microbial metabolites, tumor microenvironment, microbiome-targeted therapy, precision immuno-oncology

## Abstract

Background/Objectives: Microsatellite-stable colorectal cancer (MSS CRC) accounts for the vast majority of CRC cases and remains largely resistant to immune checkpoint inhibitors. Emerging evidence suggests that the gut microbiome is an important regulator of antitumor immunity and may contribute to immunotherapy resistance through multiple mechanisms involving the tumor microenvironment. This review aims to summarize current knowledge of the microbiome–immunity–therapy axis in MSS CRC and to explore microbiome-based strategies to enhance immunotherapy responsiveness. Methods: A narrative review of the recent literature was conducted, focusing on studies published within the last five years that investigated gut microbiota composition, microbial metabolites, tumor immune regulation, immunotherapy response, and microbiome-targeted therapeutic interventions in CRC. Evidence from mechanistic studies, translational research, clinical investigations, and multi-omics analyses was integrated. Results: Current evidence indicates that gut dysbiosis contributes to immune resistance in MSS CRC through immune exclusion, myeloid-driven immunosuppression, T-cell dysfunction, chronic inflammation, and altered microbial metabolite signaling. Specific microorganisms, including *Fusobacterium nucleatum*, enterotoxigenic *Bacteroides fragilis*, pks-positive *Escherichia coli*, and other CRC-associated pathobionts, have been implicated in tumor progression and modulation of antitumor immunity. Microbial metabolites such as short-chain fatty acids, tryptophan-derived compounds, bile acids, succinate, and inosine represent key functional mediators linking microbial communities to host immune responses. Emerging microbiome-targeted interventions, including fecal microbiota transplantation, next-generation probiotics, postbiotics, selective microbial depletion, and engineered bacterial therapeutics, have shown promising results in preclinical models and early translational or clinical studies, although robust clinical evidence remains limited. In parallel, advances in metagenomics, metabolomics, spatial transcriptomics, and artificial intelligence are facilitating the development of precision immuno-microbiome oncology approaches. Conclusions: The gut microbiome functions as a critical regulator of immune resistance in MSS CRC through coordinated effects on microbial composition, metabolite production, and tumor immune remodeling. Microbiome-targeted interventions, combined with multi-omics-based patient stratification, may provide new opportunities to overcome immunotherapy resistance and expand the clinical benefits of immune checkpoint blockade in this traditionally refractory disease.

## Simple Summary

Colorectal cancer (CRC) is one of the leading causes of cancer-related death worldwide. Although immunotherapy has transformed the treatment of several malignancies, most patients with CRC do not benefit from immune checkpoint inhibitors because their tumors belong to the microsatellite-stable (MSS) subtype, which is characterized by a weak antitumor immune response. Increasing evidence suggests that the gut microbiome plays an important role in shaping tumor immunity and influencing treatment outcomes. This review summarizes current knowledge of how alterations in gut microbial communities contribute to immunosuppression and resistance to immunotherapy in MSS CRC. We also discuss microbial metabolites, emerging microbiome-based therapeutic approaches, and recent advances in multi-omics technologies and artificial intelligence. Understanding the interactions among the microbiome, the immune system, and cancer may help identify new therapeutic strategies to improve immunotherapy responsiveness and expand treatment options for patients with CRC.

## 1. Introduction

Colorectal cancer (CRC) remains a major global oncological burden, with more than 1.9 million new cases and over 900,000 deaths estimated worldwide in 2022, ranking among the most common and lethal malignancies [1]. Despite progress in screening, surgery, chemotherapy, targeted therapy, and molecular stratification, metastatic CRC continues to be associated with poor long-term outcomes, particularly in patients who do not benefit from immune checkpoint inhibitors (ICIs) [1,2].

The therapeutic impact of immunotherapy in CRC is strongly dependent on mismatch repair status. In microsatellite instability-high/deficient mismatch repair (MSI-H/dMMR) CRC, programmed cell death protein 1 (PD-1) blockade has demonstrated durable clinical benefit, as shown by KEYNOTE-177, in which pembrolizumab improved progression-free survival compared with chemotherapy, and by CheckMate-142, in which nivolumab plus low-dose ipilimumab showed sustained activity in MSI-H/dMMR disease [3,4,5]. However, this benefit is largely restricted to a molecularly defined minority of CRC patients. In contrast, microsatellite-stable/proficient mismatch repair (MSS/pMMR) tumors represent the predominant CRC subtype and remain largely refractory to ICI monotherapy [6,7,8].

This therapeutic gap reflects fundamental biological differences between MSI-H/dMMR and MSS/pMMR tumors. MSI-H tumors generally display high mutational burden, increased neoantigen load, abundant immune infiltration, and an immune-active tumor microenvironment. Conversely, MSS CRC is typically characterized by reduced tumor immunogenicity, limited cytotoxic T-cell infiltration, immune exclusion, myeloid-driven immunosuppression, stromal barriers, and activation of oncogenic and inflammatory pathways that collectively maintain an immunologically “cold” phenotype [6,8,9].

In recent years, the gut microbiome has emerged as a critical regulator of antitumor immunity and immunotherapy response. Microbial communities can influence immune surveillance through direct interaction with epithelial and immune cells, modulation of antigen presentation, regulation of cytokine signaling, and production of immunologically active metabolites. Recent systematic and mechanistic reviews suggest that microbiota composition may contribute to differential ICI responses in CRC and other malignancies, supporting the concept of a microbiome–immunotherapy axis [10,11,12].

In CRC, dysbiosis is particularly relevant because the tumor arises within a microbe-rich anatomical environment. Enrichment of potentially pro-tumorigenic bacteria such as *Fusobacterium nucleatum* (*F. nucleatum)*, enterotoxigenic *Bacteroides fragilis*, and pks-positive *Escherichia coli* (*E. coli)*, together with depletion of beneficial short-chain fatty acid-producing commensals, may promote chronic inflammation, epithelial barrier dysfunction, immune suppression, and therapeutic resistance. Among these microorganisms, *F. nucleatum* has attracted particular attention because of its ability to modulate T-cell and natural killer cell activity, promote immune escape, and potentially reduce immunotherapy responsiveness in MSS CRC [13].

Immune resistance in MSS CRC is a multifactorial process resulting from the interplay between tumor-intrinsic molecular alterations and host-related factors. Besides the low tumor mutational burden and limited neoantigen landscape characteristic of MSS tumors, oncogenic signaling pathways, particularly KRAS mutations, together with aberrant WNT/β-catenin and transforming growth factor beta (TGF-β) signaling, contribute to the establishment of an immunosuppressive tumor microenvironment by limiting cytotoxic T-cell infiltration, promoting myeloid cell recruitment, impairing antigen presentation, and facilitating immune evasion. Emerging evidence indicates that the gut microbiome represents an additional regulatory layer that interacts with these tumor-intrinsic mechanisms through microbial metabolites, immune modulation, and host–microbe interactions, thereby shaping and reinforcing the immune-resistant phenotype rather than acting as its sole determinant [14].

**Figure 1:**
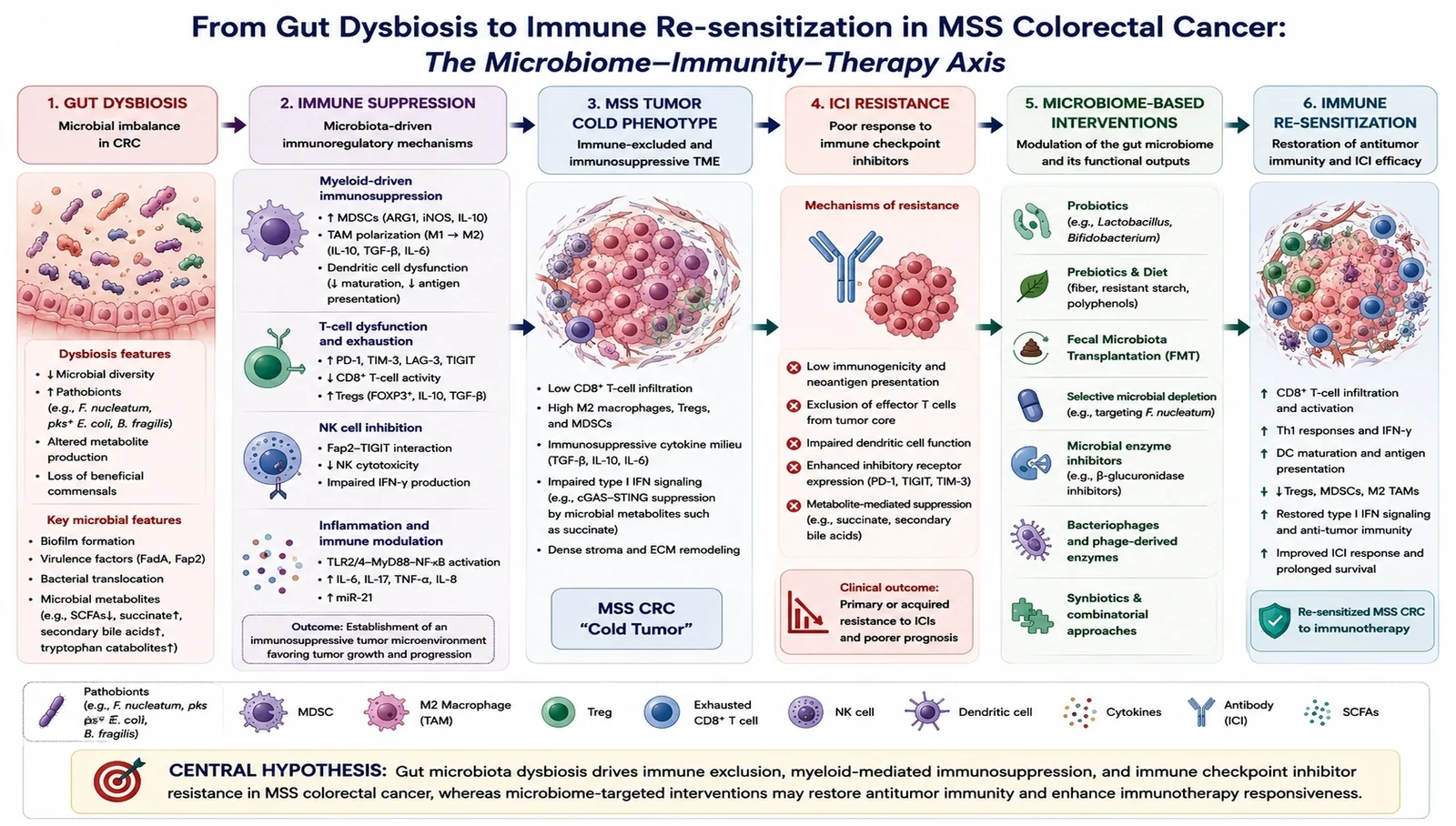
Proposed role of the gut microbiome in shaping immune resistance and therapeutic responsiveness in microsatellite-stable colorectal cancer. Graphical illustration generated with the assistance of ChatGPT (OpenAI, GPT-5.5) based on detailed author-designed scientific concepts and subsequently reviewed and approved by the authors. Dysbiosis-associated immune suppression, microbial metabolites, and tumor microenvironment remodeling contribute to immune checkpoint inhibitor resistance, whereas microbiome-targeted interventions may promote immune reactivation and therapeutic re-sensitization.

Therefore, the central hypothesis of this review is that gut microbiome dysbiosis represents an important regulator of the immune-resistant phenotype of MSS CRC by interacting with tumor-intrinsic oncogenic pathways, promoting immune exclusion, myeloid-driven immunosuppression, T-cell dysfunction, altered microbial metabolite signaling, and reduced responsiveness to immune checkpoint inhibitors. These processes collectively promote resistance to immune checkpoint inhibitors and help maintain the immunologically “cold” tumor microenvironment characteristic of MSS CRC. We further propose that microbiome-targeted interventions—including microbial modulation, metabolite-based therapies, fecal microbiota transplantation, engineered bacterial therapeutics, and precision multi-omics approaches—may represent rational strategies to restore antitumor immunity and enhance immunotherapy responsiveness in this traditionally refractory CRC subtype. Based on this conceptual framework, this review examines the mechanistic links between gut dysbiosis, immune resistance, and therapeutic failure in MSS CRC, while highlighting emerging opportunities for microbiome-guided precision immuno-oncology. These concepts are integrated and summarized in Figure 1.

## 2. Literature Search Strategy and Review Methodology

### 2.1. Literature Search and Evidence Synthesis

This narrative review was conducted to provide an up-to-date synthesis of evidence on the interactions among the gut microbiome, antitumor immunity, and immunotherapy resistance in MSS CRC. A structured literature search was performed in PubMed/MEDLINE, Scopus, and Web of Science, focusing primarily on studies published between January 2020 and May 2026.

The search strategy combined Medical Subject Headings (MeSH) terms and free-text keywords related to colorectal cancer, gut microbiota, immunotherapy, immune checkpoint inhibitors, microbial metabolites, tumor microenvironment, fecal microbiota transplantation, probiotics, postbiotics, engineered bacterial therapeutics, multi-omics, and artificial intelligence. Reference lists of relevant reviews and original studies were also manually screened to identify additional eligible publications.

Priority was given to recent original research articles, systematic reviews, meta-analyses, and high-quality translational studies published in peer-reviewed journals. Landmark studies published before 2020 were included when they provided essential mechanistic or clinical evidence that remains fundamental for understanding the microbiome–immunity–therapy axis, such as pivotal studies on immune checkpoint inhibitors or the role of microbial genotoxins in colorectal carcinogenesis.

Evidence was synthesized narratively, with particular emphasis on mechanistic insights linking gut dysbiosis to immune exclusion, myeloid-driven immunosuppression, microbial metabolite signaling, and resistance to immune checkpoint inhibitors in MSS CRC. Special attention was also given to emerging microbiome-targeted therapeutic strategies, including fecal microbiota transplantation, probiotics, postbiotics, engineered bacterial therapeutics, and precision multi-omics approaches that may improve immunotherapy responsiveness.

This review was conducted as a narrative review and therefore did not follow the Preferred Reporting Items for Systematic Reviews and Meta-Analyses (PRISMA) statement.

### 2.2. Use of Generative Artificial Intelligence

**Figure 2:**
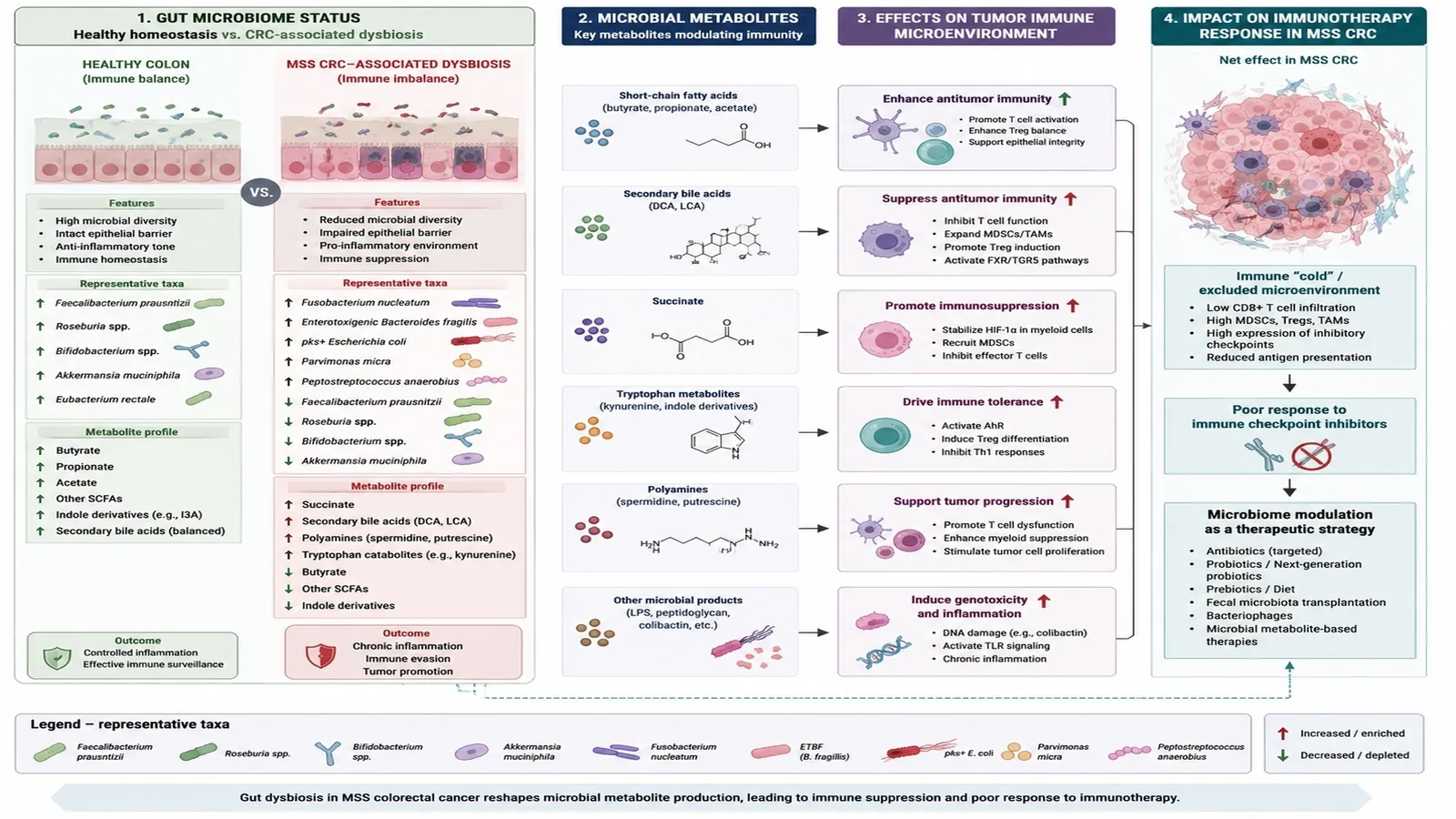
Gut microbiome dysbiosis, microbial metabolites, and immune regulation in microsatellite-stable colorectal cancer (MSS CRC). Graphical illustration generated with the assistance of ChatGPT (OpenAI, GPT-5.5) based on detailed author-designed scientific concepts and subsequently reviewed and approved by the authors. The figure compares the microbial composition and metabolic profiles of a healthy colon and MSS CRC-associated dysbiosis, highlighting representative bacterial taxa that are enriched or depleted during disease progression. Alterations in microbial metabolites, including short-chain fatty acids, secondary bile acids, succinate, tryptophan-derived metabolites, polyamines, and other microbial products, modulate antitumor immunity by influencing dendritic-cell function, macrophage polarization, myeloid-derived suppressor cells, regulatory T cells, and cytotoxic T-cell activity. Collectively, these mechanisms promote an immune-excluded, immunosuppressive tumor microenvironment that contributes to reduced responsiveness to ICIs. The figure also summarizes potential microbiome-targeted therapeutic strategies to restore antitumor immunity in MSS CRC.

**Figure 3:**
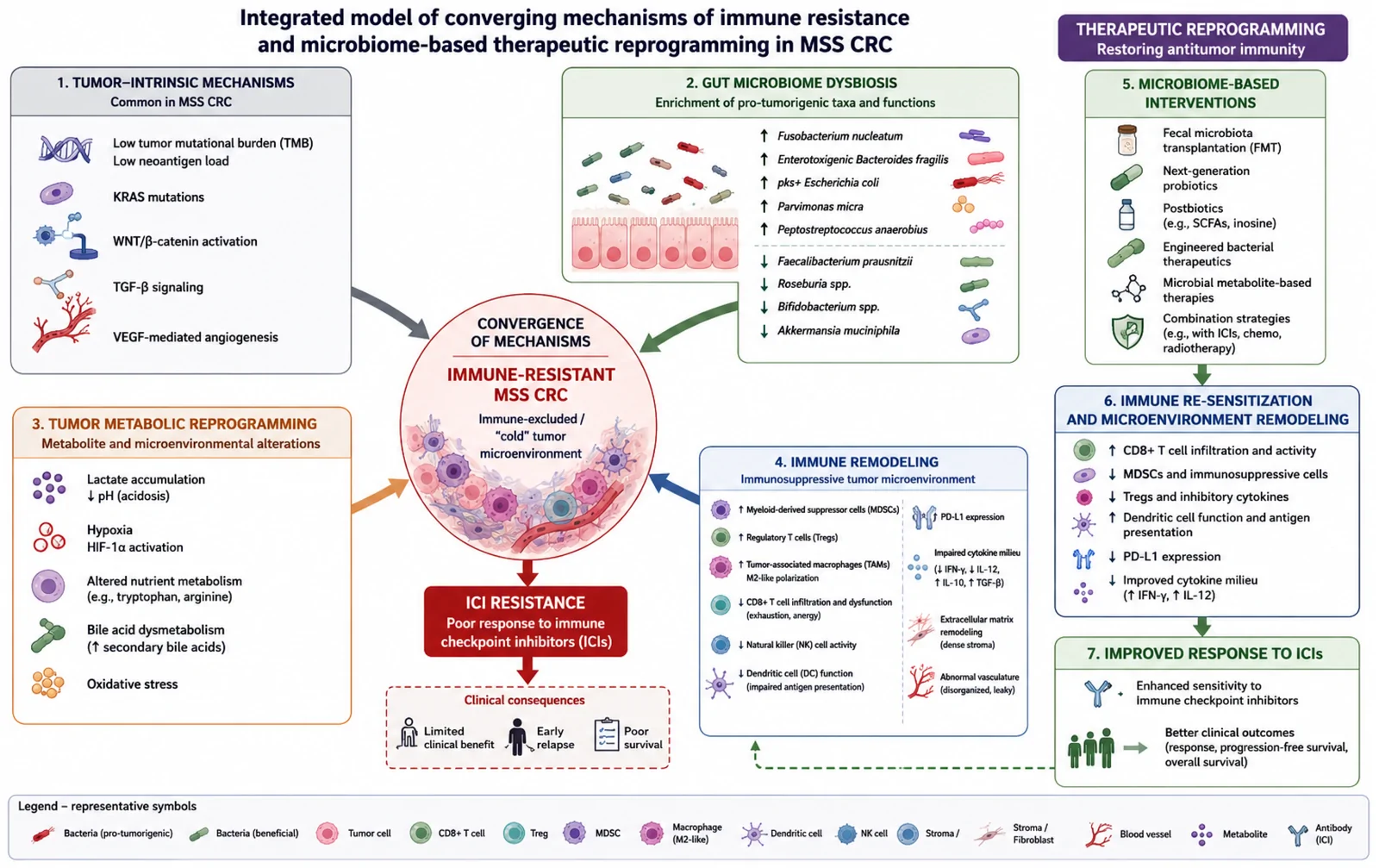
Integrated Model of Converging Mechanisms Driving Immune Resistance and Microbiome-Based Therapeutic Reprogramming in Microsatellite-Stable Colorectal Cancer (MSS CRC). Graphical illustration generated with the assistance of ChatGPT (OpenAI, GPT-5.5) based on detailed author-designed scientific concepts and subsequently reviewed and approved by the authors. Integrated model illustrating how tumor-intrinsic molecular alterations, metabolic remodeling, gut microbiome dysbiosis, and immune-cell dysfunction converge to establish the immune-resistant phenotype characteristic of MSS CRC. These interconnected mechanisms collectively promote immune exclusion and resistance to immune checkpoint inhibitors (ICIs). Microbiome-targeted therapeutic strategies, including fecal microbiota transplantation, next-generation probiotics, postbiotics, engineered bacterial therapeutics, and metabolite-based interventions, may remodel the tumor immune microenvironment, restore antitumor immunity, and improve responsiveness to ICIs. Upward arrows (↑) indicate increased expression, activation, or pathological enhancement, whereas downward arrows (↓) denote decreased function, depletion, or impairment.

Generative artificial intelligence (GenAI), specifically ChatGPT (OpenAI, GPT-5.5), was used exclusively to assist in the preparation of the graphical illustrations included in this review (Figure 1, Figure 2 and Figure 3). The authors conceived the scientific concepts, biological mechanisms, figure organization, and visual content of each figure and provided detailed iterative instructions to generate graphical renderings that accurately reflected the evidence synthesized in the manuscript. The AI tool was used solely for visual illustration and graphical rendering and was not used for literature searching, study selection, evidence synthesis, data extraction, data analysis, interpretation of findings, or manuscript writing. All graphical outputs were critically reviewed, verified, and approved by the authors, who take full responsibility for the scientific accuracy, integrity, and final content of the figures and the manuscript.

## 3. Microsatellite-Stable Colorectal Cancer as an Immunologically Cold Tumor

### 3.1. Reduced Antigenicity and Neoantigen Burden

MSS/pMMR colorectal cancer is characterized by relatively low intrinsic immunogenicity, representing one of the principal biological mechanisms underlying its poor responsiveness to ICIs. Compared with MSI-H/dMMR tumors, MSS CRC generally exhibits a lower tumor mutational burden (TMB) and consequently generates fewer neoantigens that can be recognized by the adaptive immune system [3,6,8]. This reduced neoantigenic landscape limits efficient dendritic-cell-mediated T-cell priming and diminishes the likelihood of generating robust cytotoxic CD8^+^ T-cell responses. As a result, PD-1/programmed death-ligand 1 (PD-L1) blockade may relieve inhibitory immune signaling but often fails to induce clinically meaningful antitumor immunity in the absence of pre-existing immune activation [6,7,8].

In contrast, MSI-H/dMMR colorectal cancers accumulate numerous insertion–deletion mutations owing to defective DNA mismatch repair, resulting in a substantially higher tumor mutational burden and the generation of abundant neoantigens [3,6,8]. This highly immunogenic phenotype promotes efficient immune recognition, increased infiltration of activated CD8^+^ T lymphocytes, enhanced interferon signaling, and elevated expression of immune checkpoint molecules. These biological characteristics explain, at least in part, the durable clinical responses observed with pembrolizumab, nivolumab, and nivolumab–ipilimumab in patients with MSI-H/dMMR CRC [3,4,5].

### 3.2. Impairment of the Cancer–Immunity Cycle and Immune Exclusion

Effective antitumor immunity depends on the successful completion of the cancer–immunity cycle, including antigen uptake by dendritic cells, migration to lymphoid structures, presentation to naïve T cells, expansion of tumor-specific cytotoxic lymphocytes, and trafficking of these cells into tumor tissue. In MSS CRC, several steps of this coordinated process may be impaired, ultimately resulting in ineffective T-cell activation, immune exclusion, and limited lymphocyte infiltration into the tumor core [8,9].

Many MSS tumors show an immune-excluded phenotype, in which lymphocytes are present at the invasive margin but fail to penetrate the tumor parenchyma. This spatial immune exclusion limits direct contact between CD8+ T cells and malignant epithelial cells, reducing the efficacy of checkpoint inhibition. Tumor-intrinsic pathways, including Wnt/β-catenin, MAPK, PI3K–AKT, and KRAS-driven signaling, may contribute to defective dendritic-cell recruitment, reduced T-cell infiltration, and immune escape [6,8,9].

### 3.3. Myeloid-Driven Immunosuppression

The immunosuppressive myeloid compartment is a central determinant of immune resistance in MSS CRC. Tumor-associated macrophages, myeloid-derived suppressor cells, neutrophils, and immature dendritic cell populations can suppress cytotoxic T-cell activity through multiple mechanisms, including arginine depletion, reactive oxygen species production, secretion of interleukin-10 (IL-10) and TGF-β signaling, induction of regulatory T cells, and inhibition of antigen presentation [8,9].

Tumor-associated macrophages may acquire an M2-like phenotype that supports angiogenesis, matrix remodeling, tumor invasion, and immune evasion. Similarly, myeloid-derived suppressor cells in colorectal cancer (MDSCs) accumulate in the CRC microenvironment and inhibit T-cell proliferation and effector function. These myeloid populations may also sustain resistance to ICIs by maintaining a suppressive cytokine network that cannot be reversed by PD-1/PD-L1 blockade alone [8,9].

### 3.4. Stromal and Vascular Barriers

The stromal architecture of MSS CRC further contributes to its cold immune phenotype. Cancer-associated fibroblasts, extracellular matrix remodeling, and abnormal vasculature create both physical and biochemical barriers to immune-cell infiltration. TGF-β signaling is particularly relevant because it promotes fibroblast activation, immune exclusion, epithelial–mesenchymal transition, and suppression of effector T-cell function [8,9].

Vascular endothelial growth factor (VEGF)-mediated abnormal angiogenesis also restricts immune cell trafficking and promotes an immunosuppressive microenvironment. These stromal and vascular barriers help explain why many MSS tumors remain resistant even when checkpoint molecules are therapeutically inhibited. Consequently, combination strategies aimed at vascular normalization, stromal remodeling, or TGF-β inhibition are being explored as potential approaches to sensitize MSS CRC to immunotherapy [6,8].

### 3.5. Tumor Metabolic Reprogramming: Lactate, Hypoxia, and Acidosis as Drivers of Immune Suppression

Tumor metabolic reprogramming has emerged as a critical determinant of immune resistance in CRC, providing a mechanistic link between tumor-intrinsic biology, the immune microenvironment, and the gut microbiome. As rapidly proliferating tumor cells undergo aerobic glycolysis (the Warburg effect), excessive lactate accumulates in the tumor microenvironment, leading to extracellular acidification, nutrient competition, and tissue hypoxia. These metabolic alterations profoundly influence both innate and adaptive immune responses and contribute to the establishment of an immunologically cold phenotype that is characteristic of MSS CRC [15,16].

Lactate is now recognized as an active immunoregulatory metabolite rather than a simple by-product of glycolysis. Elevated lactate concentrations suppress dendritic-cell maturation, impair antigen presentation, inhibit CD8^+^ cytotoxic T-cell proliferation and interferon-γ production, reduce natural killer cell cytotoxicity, and promote polarization of tumor-associated macrophages toward an immunosuppressive M2 phenotype. Lactate accumulation also promotes the expansion of regulatory T cells and myeloid-derived suppressor cells, thereby reinforcing immunosuppression through multiple complementary mechanisms. In CRC, increased lactate production has been associated with enhanced tumor progression, immune evasion, and resistance to anticancer therapies [15,16].

Hypoxia further amplifies these immunosuppressive effects by stabilizing hypoxia-inducible factor-1α (HIF-1α). Activation of HIF-1α promotes glycolytic metabolism, increases lactate production, upregulates VEGF, and enhances PD-L1 expression on both tumor and immune cells. Collectively, these alterations reduce cytotoxic T-cell infiltration, impair dendritic cell function, and promote the accumulation of immunosuppressive myeloid populations within the tumor microenvironment. Persistent hypoxia also contributes to abnormal angiogenesis and stromal remodeling, creating additional physical barriers that limit immune-cell trafficking into the tumor core [16,17].

Extracellular acidosis represents another hallmark of the metabolically reprogrammed tumor microenvironment. Acidic pH compromises T-cell receptor signaling, decreases cytokine secretion, suppresses natural killer cell activity, and limits the metabolic fitness of effector lymphocytes. Simultaneously, acidic conditions promote tumor cell survival, extracellular matrix remodeling, and invasive behavior, further contributing to immune escape and therapeutic resistance. These findings indicate that metabolic remodeling is not merely a consequence of tumor progression but an active regulator of antitumor immunity [15,16].

Importantly, tumor metabolism and the gut microbiome are increasingly recognized as interconnected biological systems. Tumor-derived metabolites, particularly lactate, alter the intestinal and local tumor microenvironment, influencing microbial composition and metabolic activity. Conversely, gut microbial metabolites—including short-chain fatty acids, secondary bile acids, tryptophan-derived metabolites, succinate, and inosine—modulate immune-cell differentiation, inflammatory signaling, and cellular metabolism, thereby interacting with tumor-derived metabolic pathways. Rather than functioning as independent processes, tumor metabolism and microbiome-derived signaling form an integrated metabolic–immune network that collectively shapes immune responsiveness in MSS CRC [17,18].

These observations provide an important mechanistic bridge between tumor-intrinsic metabolic remodeling and the immunomodulatory effects of the gut microbiome discussed in the following section. They also suggest that combining metabolic interventions with microbiome-targeted strategies and immune checkpoint inhibitors may represent a rational therapeutic approach for overcoming immune resistance in MSS CRC [16,17].

### 3.6. The Gut Microbiome as an Additional Layer of Immune Resistance

Although tumor-intrinsic, stromal, vascular, and metabolic mechanisms are central contributors to immune resistance in MSS CRC, the gut microbiome represents an additional regulatory layer that can interact with each of these processes. The microbiome can influence the tumor immune microenvironment through microbial antigens, pathogen-associated molecular patterns, microbial metabolites, and modulation of systemic inflammatory tone [10,11,12]. In CRC, this relationship is particularly relevant because dysbiotic microbial communities are located in direct proximity to the tumor.

Certain bacteria may promote immune escape by suppressing cytotoxic lymphocytes, polarizing macrophages, expanding MDSCs, or increasing checkpoint molecule expression. *F. nucleatum* is a model organism in this context, as recent evidence links it to immune modulation, reduced cytotoxic activity of T and NK cells, and possible impairment of immunotherapy responsiveness in MSS CRC [13]. These observations suggest that although host–microbe interactions influence tumor biology across the colorectal cancer spectrum, they may play a particularly important role in shaping the immune-resistant phenotype of MSS CRC, in which microbial modulation represents an additional regulatory layer superimposed on tumor-intrinsic and stromal mechanisms of immune evasion.

## 4. *Fusobacterium nucleatum* as a Key Contributor to Immune Resistance in MSS Colorectal Cancer

Among CRC-associated microorganisms, *F. nucleatum* has emerged as one of the most extensively investigated bacterial species linking gut dysbiosis, tumor progression, immune remodeling, and therapeutic resistance. Current evidence indicates that its biological effects are multifaceted and can be broadly categorized into three complementary domains: (i) tumor-promoting activities, including epithelial adhesion and activation of oncogenic signaling pathways; (ii) immune-suppressive effects that contribute to immune evasion and resistance to immunotherapy; and (iii) context-dependent interactions that vary according to molecular subtype, bacterial strain, microbial burden, and the host immune microenvironment. Although these mechanisms frequently overlap, distinguishing them facilitates interpretation of the current evidence regarding the role of *F. nucleatum* in CRC [13,19,20].

Because the distribution and biological significance of *F. nucleatum* vary across CRC molecular subtypes, its role in MSS disease warrants careful consideration. Current evidence indicates that although *F. nucleatum* is generally more abundant in proximal MSI-H tumors, it is also present in a substantial proportion of MSS CRCs, for which growing evidence supports its contribution to immune modulation and therapeutic resistance. This distinction is discussed below.

### 4.1. Distribution of Fusobacterium nucleatum Across Molecular Subtypes of Colorectal Cancer

*F. nucleatum* is among the bacterial species most consistently associated with CRC; however, its distribution is not uniform across molecular subtypes. Early microbiome studies reported a preferential enrichment of *F. nucleatum* in proximal colon cancers, which are frequently characterized by microsatellite instability (MSI-H/dMMR), CpG island methylator phenotype (CIMP-high), and BRAF mutations. These observations initially suggested a close association between *F. nucleatum* colonization and the MSI-H molecular subtype [13,19,20].

Subsequent studies have demonstrated that *F. nucleatum* is not restricted to MSI-H tumors but is also detectable in a substantial proportion of MSS/pMMR CRCs, although generally at lower abundance. Increasing evidence indicates that bacterial load alone does not fully determine biological relevance. Rather, strain-specific virulence factors, host immune status, tumor location, and interactions with the surrounding microbial ecosystem collectively influence the biological effects of *F. nucleatum* within the tumor microenvironment [13,19,20,21].

Importantly, recent translational studies suggest that the role of *F. nucleatum* in MSS CRC is more complex than previously appreciated. While numerous investigations have associated *F. nucleatum* with chronic inflammation, immunosuppression, and therapeutic resistance, emerging data indicate that, under specific biological contexts, intratumoral F. nucleatum may also influence immune cell activation and responsiveness to immune checkpoint blockade. These apparently conflicting observations likely reflect differences in bacterial strains, tumor molecular characteristics, microbial burden, spatial localization within the tumor, and the baseline immune landscape rather than fundamentally opposing biological functions [21,22].

Therefore, although *F. nucleatum* is generally more abundant in proximal MSI-H CRC, current evidence supports its biological relevance across the colorectal cancer spectrum, including MSS CRC. In this setting, *F. nucleatum* should be viewed as an important contributor to immune remodeling rather than a microorganism exclusively associated with MSI-H disease, with its effects depending on the molecular and immunological context of the host tumor [13,19,20,21,22].

### 4.2. Adhesion, Epithelial Invasion, and Oncogenic Signaling

The pro-tumorigenic effects of *F. nucleatum* begin with its ability to adhere to and invade colorectal epithelial cells. The bacterial adhesin FadA binds to E-cadherin, disrupts epithelial junction integrity, and activates Wnt/β-catenin signaling, a key oncogenic pathway in CRC. This interaction promotes epithelial proliferation, survival, epithelial–mesenchymal transition, and invasive behavior, thereby linking microbial colonization to canonical CRC oncogenic signaling [19,20].

In addition to FadA, *F. nucleatum* expresses Fap2, an autotransporter protein that recognizes Gal-GalNAc residues enriched on colorectal tumor cells. This selective tumor tropism may explain why *F. nucleatum* preferentially accumulates in tumor tissue. Fap2 is also immunologically relevant because it can interact with TIGIT, an inhibitory receptor expressed on natural killer cells and T cells, thereby reducing antitumor cytotoxicity [13,19].

### 4.3. Inflammation-Driven Immune Remodeling

A major mechanism through which *F. nucleatum* contributes to CRC progression is the induction of chronic inflammation. The bacterium activates innate immune receptors, including TLR2 and TLR4, leading to downstream NF-κB and MyD88 signaling and increased production of inflammatory mediators, including IL-6, IL-8, IL-17, tumor necrosis factor α (TNF-α), and other cytokines involved in tumor progression [19,20].

This inflammatory activity is clinically relevant because chronic cytokine signaling supports epithelial proliferation, angiogenesis, tumor invasion, and immune tolerance. Recent reviews emphasize that *F. nucleatum* can create a tumor-promoting inflammatory niche while simultaneously impairing antitumor immune responses, a combination that may be particularly relevant in MSS CRC, where the immune microenvironment is already poorly responsive to ICI monotherapy [13,19,20].

### 4.4. Suppression of Cytotoxic T-Cell and NK-Cell Activity

Beyond inflammation, *F. nucleatum* directly contributes to immune escape. One of the best-described mechanisms involves Fap2-mediated interaction with TIGIT, which suppresses NK-cell cytotoxicity and reduces T-cell-mediated antitumor immunity [13,19]. This mechanism is highly relevant in MSS CRC, where baseline cytotoxic T-cell infiltration and immune activation are often limited.

Recent studies and reviews also suggest that *F. nucleatum* can modulate the tumor immune microenvironment more broadly, including effects on T-cell dysfunction, macrophage polarization, and myeloid-driven immunosuppression [13,21]. These effects may reinforce the immune-excluded or immune-suppressed phenotype characteristic of many MSS tumors.

### 4.5. F. nucleatum, Interferon Signaling, and PD-1 Resistance

One of the most important recent developments is the proposed link between *F. nucleatum*, microbial metabolites, and impaired response to PD-1 blockade. In MSS CRC models, *F. nucleatum* has been reported to influence anti-PD-1 responsiveness, indicating that its role in immunotherapy may be context-dependent rather than uniformly suppressive [22]. At the same time, other mechanistic evidence suggests that *F. nucleatum*-derived metabolites, including succinic acid, may impair cGAS–STING–IFN-β signaling, reduce dendritic-cell activation, and compromise antitumor immune priming [13].

This duality is important for the review. *F. nucleatum* should not be presented simplistically as only “bad” or only “pro-resistance”; rather, it appears to be a context-dependent microbial regulator of the tumor immune microenvironment. Its effects may vary depending on bacterial strain, tumor molecular subtype, metabolite production, immune baseline, and therapeutic combination.

### 4.6. Therapeutic Implications

Because *F. nucleatum* is linked to immune suppression, tumor progression, and therapy resistance, it represents a rational therapeutic target. Potential strategies include selective microbial depletion, bacteriophage-based approaches, dietary modulation, nanodrug delivery systems, and microbiome-based patient stratification [13,23]. However, current evidence remains largely preclinical or observational, and no interventional clinical trial has yet proven that direct targeting of *F. nucleatum* improves immunotherapy outcomes in MSS CRC.

Therefore, *F. nucleatum* should currently be viewed as a translationally promising but not yet clinically validated target. Its strongest immediate value may lie in biomarker development, especially for identifying patients with microbiome-driven immune suppression who may benefit from microbiome-modulating strategies combined with immunotherapy.

## 5. Beyond *Fusobacterium nucleatum*: Other Microbial Contributors to Immune Resistance in MSS Colorectal Cancer

Although *F. nucleatum* is the most extensively studied CRC-associated bacterium, immune resistance in MSS CRC is unlikely to be driven by a single microorganism. Current evidence supports a broader dysbiotic ecosystem model, in which multiple microbial taxa, biofilm communities, bacterial genotoxins, and microbial metabolites interact with epithelial, stromal, and immune compartments to shape the tumor microenvironment [24,25].

Recent microbiome studies show that CRC-associated dysbiosis is characterized by enrichment of pathobionts such as enterotoxigenic *Bacteroides fragilis*, pks-positive *E. coli*, *Parvimonas micra*, *Peptostreptococcus anaerobius*, and other oral or intestinal anaerobes, together with depletion of protective short-chain fatty acid-producing commensals [24,25]. This suggests that microbiome-associated immune resistance should be interpreted as a network-level process rather than the effect of isolated bacterial species.

### 5.1. Enterotoxigenic Bacteroides fragilis: Inflammation and Immune Dysregulation

Enterotoxigenic *Bacteroides fragilis* (ETBF) is one of the best-characterized pro-tumorigenic bacteria in CRC. ETBF produces *Bacteroides fragilis* toxin, also known as fragilysin, which disrupts epithelial barrier integrity, activates β-catenin and STAT3-related signaling, and promotes chronic inflammation [24,25,26]. ETBF-driven inflammation is particularly relevant because it can induce IL-17- and IL-6-rich immune responses that support tumor growth, myeloid recruitment, and immunosuppression.

A recent systematic review and meta-analysis reported that ETBF is associated with CRC and may vary across stages of colorectal carcinogenesis [26]. Additional recent data suggest that ETBF may be linked to immune modulation in CRC liver metastasis, supporting the idea that this bacterium can shape not only primary tumor biology but also metastatic immune niches [27].

Unlike *F. nucleatum*, which directly interferes with cytotoxic immune mechanisms, ETBF appears to promote immune resistance mainly through chronic inflammatory signaling, barrier disruption, and myeloid-promoting cytokine networks. These mechanisms may cooperate with other microbial drivers to sustain the immune-suppressive MSS CRC microenvironment.

### 5.2. pks-Positive Escherichia coli: Genotoxicity and Tumor Evolution

pks-positive *E. coli* carries the polyketide synthase genomic island responsible for the production of colibactin, a genotoxic compound capable of inducing DNA damage. Colibactin exposure produces characteristic mutational signatures in colorectal tumors, providing one of the strongest causal links between a microbial product and human CRC genome alteration [28,29].

Recent evidence has further strengthened this concept by demonstrating that exposure to *pks*-positive *E. coli* during early childhood may leave persistent colibactin-associated mutational signatures that remain detectable decades later in patients with early-onset colorectal cancer. These findings support the hypothesis that early-life microbial exposure, potentially influenced by Western dietary habits, antibiotic use, and lifestyle-related alterations of the gut microbiome, may initiate carcinogenic processes long before clinical disease becomes apparent. This paradigm extends the role of *pks*-positive *E. coli* beyond tumor progression, suggesting that microbial-induced genomic damage may represent one of the earliest events in colorectal carcinogenesis [30]. Although the landmark organoid and mutational signature studies were published before the last five-year window, they remain mechanistically essential and should be retained as foundational references [28,29]. More recent integrative reviews continue to highlight pks-positive *E. coli* as a key microbial driver of CRC through genotoxicity, disruption of the epithelial barrier, inflammation, and tumor evolution [24,25].

From the perspective of immunotherapy resistance, pks-positive *E. coli* may contribute indirectly by accelerating genomic instability, epithelial stress, inflammatory signaling, and tumor heterogeneity. Together, these observations suggest that the oncogenic consequences of pks-positive *E. coli* may begin long before tumor diagnosis, thereby providing a mechanistic link between early microbial exposure, genomic instability, and subsequent immune evolution during colorectal carcinogenesis.

### 5.3. Parvimonas micra, Peptostreptococcus anaerobius, and Emerging Pathobionts

In addition to ETBF and pks-positive *E. coli*, several oral and intestinal anaerobes have been associated with CRC development and progression. *P. micra* has been repeatedly identified as enriched in CRC-associated microbial signatures and has been shown experimentally to promote colorectal tumorigenesis [31]. Recent evidence also suggests that *P. micra* may contribute to carcinogenesis through microbial metabolite-mediated DNA damage [32].

*P. anaerobius* is another CRC-associated pathobiont with potential immunological relevance. It has been reported to promote colorectal carcinogenesis and modulate the tumor immune microenvironment through PI3K/Akt-related signaling and inflammatory mechanisms [33]. Although the strongest mechanistic study is more than 5 years old, recent reviews continue to recognize *P. anaerobius* as part of the CRC-associated dysbiotic network [24,25].

These organisms are important because they support the view that CRC-associated dysbiosis is not a single-pathogen phenomenon. Instead, several bacterial taxa may act through complementary mechanisms: adhesion, inflammation, immune modulation, metabolite production, and genotoxicity.

### 5.4. Biofilms as Immunomodulatory Ecosystems

Bacterial biofilms may represent an underappreciated determinant of immune resistance in CRC. Biofilms are organized microbial communities embedded in extracellular matrices, allowing microbial persistence, spatial organization, and coordinated interactions with host tissues.

A 2024 study using fluorescence in situ hybridization and dual-RNA sequencing showed that biofilms and core pathogens shape the CRC tumor microenvironment and immune phenotype. The study reported that *Fusobacterium* spp. were associated with increased bacterial biomass and an inflammatory response, while high bacterial activity correlated with pro-inflammatory cytokines, matrix-remodeling factors, immunomodulatory molecules, M2 macrophages, regulatory T cells, and other immune cell populations [34].

These findings are highly relevant because they move the field beyond static microbiome composition. They suggest that bacterial activity, spatial organization, and biofilm biology may be more informative than the presence of individual taxa alone. In MSS CRC, such microbial ecosystems may sustain chronic inflammation while promoting immune tolerance, thereby contributing to immune exclusion and ICI resistance.

### 5.5. A Microbial Network Model of Immune Resistance

Collectively, current evidence supports a transition from a pathogen-centric model to a network-based model of microbiome-driven immune resistance. In this framework, *F. nucleatum*, ETBF, pks-positive *E. coli*, *P. micra*, *P. anaerobius*, and biofilm-associated microbial communities interact to promote barrier dysfunction, inflammation, immune suppression, metabolic remodeling, and tumor progression [24,25,31].

This integrated model provides the mechanistic foundation for the next section of the review. While bacterial taxa can influence CRC through direct host–microbe interactions, many of the systemic and local immunological effects of dysbiosis are mediated by microbial metabolites. These bioactive molecules serve as a functional bridge between microbial community composition and immunotherapy responsiveness in MSS CRC.

While individual bacterial taxa contribute to immune remodeling through direct host–microbe interactions, many of the systemic immunological consequences of dysbiosis are ultimately mediated by microbial metabolites. These bioactive molecules represent a functional bridge between microbial community composition and host immune responses, providing a mechanistic link between gut dysbiosis and immunotherapy responsiveness in MSS CRC.

The microorganisms discussed above contribute to colorectal carcinogenesis and immune resistance through distinct yet frequently overlapping mechanisms. To facilitate comparison of their biological activities and potential relevance to immunotherapy responsiveness, the principal CRC-associated microbial taxa and their immunological effects are summarized in Table 1.

The table summarizes the principal microbial virulence mechanisms, their effects on the tumor immune microenvironment, the proposed implications for immunotherapy responsiveness, the current level of supporting evidence, and representative references for each microorganism.

## 6. Microbial Metabolites Linking Gut Dysbiosis to Immunotherapy Resistance and Immune Re-Sensitization in MSS Colorectal Cancer

Recent advances in microbiome research have shifted attention from taxonomic composition toward microbial functionality. Although specific bacterial species are frequently associated with colorectal carcinogenesis and immune remodeling, mounting evidence indicates that many of their biological effects are mediated through the production of bioactive metabolites. These molecules function as signaling mediators between the microbiota and the host, influencing epithelial integrity, innate immunity, adaptive immune responses, and tumor progression [35,36,37].

In CRC, microbial metabolites represent a critical mechanistic bridge linking gut dysbiosis to tumor immune escape. By modulating dendritic-cell maturation, macrophage polarization, T-cell differentiation, cytokine production, and immune checkpoint signaling, these metabolites may profoundly influence the responsiveness of MSS CRC to immune checkpoint inhibitors [35,36,37,38]. Importantly, unlike bacterial composition, which varies substantially among individuals, microbial metabolites often converge into common functional pathways, making them attractive candidates for biomarker development and therapeutic intervention.

### 6.1. Short-Chain Fatty Acids: Beneficial Mediators of Antitumor Immunity

Short-chain fatty acids (SCFAs), including butyrate, propionate, and acetate, are among the most extensively studied microbiota-derived metabolites. They are generated through bacterial fermentation of dietary fibers and are primarily produced by beneficial commensal bacteria such as *Faecalibacterium prausnitzii*, *Roseburia* spp., and members of the Lachnospiraceae family [35,36].

Among SCFAs, butyrate has attracted particular interest because of its anti-inflammatory and antitumor properties. Butyrate serves as the primary energy source for colonocytes, enhances epithelial barrier integrity, and modulates immune responses by inhibiting histone deacetylases (HDACs). Through this mechanism, butyrate can influence gene expression, promote differentiation of regulatory immune populations, and regulate inflammatory signaling pathways [35,39].

Recent evidence suggests that SCFAs may also enhance antitumor immunity. Experimental studies have shown that butyrate can improve CD8+ T-cell function, support memory T-cell formation, and influence dendritic-cell activity. Moreover, SCFAs may modulate the efficacy of immune checkpoint blockade by affecting T-cell metabolism and cytokine production [36,37,38].

In CRC-associated dysbiosis, depletion of SCFA-producing bacteria frequently results in reduced butyrate availability. Consequently, loss of SCFA-mediated immune regulation may contribute to chronic inflammation, impaired barrier function, and immune dysfunction, thereby facilitating the development of the immunologically cold MSS tumor phenotype.

Nevertheless, the immunological effects of SCFAs should not be considered uniformly beneficial. Increasing evidence indicates that their biological activity is highly context-dependent and may vary according to metabolite concentration, tumor stage, host metabolic status, microbial composition, the local immune microenvironment, and the therapeutic setting. While physiological levels of butyrate generally support epithelial homeostasis and antitumor immunity, altered SCFA availability or signaling may differentially regulate immune cell subsets, including regulatory T cells and effector lymphocytes, depending on the biological context. Moreover, the impact of SCFAs may vary according to concurrent therapeutic interventions, including immune checkpoint blockade. Consequently, the role of SCFAs in MSS CRC should be interpreted within the broader framework of host–microbiome interactions rather than as universally protective or detrimental [36,37,38,39].

### 6.2. Tryptophan Metabolism and the Aryl Hydrocarbon Receptor Pathway

Tryptophan metabolism represents another major microbiome-dependent pathway with important immunological implications. Intestinal bacteria metabolize dietary tryptophan into a variety of indole derivatives that can activate the aryl hydrocarbon receptor (AhR), a ligand-dependent transcription factor involved in mucosal immunity and immune homeostasis [36,40].

AhR signaling regulates epithelial barrier maintenance, cytokine production, dendritic cell differentiation, and T cell responses. Physiological activation of AhR contributes to intestinal homeostasis and protection against excessive inflammation. However, dysregulated tryptophan metabolism may alter immune equilibrium and contribute to tumor-promoting inflammation [40,41].

Emerging evidence indicates that microbiota-derived indoles can influence antitumor immunity by regulating CD8+ T-cell activity, dendritic cell maturation, cytokine production, and the balance between effector and regulatory immune responses. Dysregulated tryptophan metabolism and aberrant AhR signaling may therefore contribute to impaired immune surveillance, reduced antigen presentation, and the establishment of an immunosuppressive tumor microenvironment that limits the efficacy of ICIs in MSS CRC [36,40].

Because AhR signaling represents a potentially druggable pathway, microbiome-mediated modulation of tryptophan metabolism has attracted increasing interest as a therapeutic strategy to enhance immunotherapy responsiveness. Targeting the tryptophan–AhR axis may improve dendritic cell function, restore cytotoxic T cell activity, and potentially enhance responsiveness to immune checkpoint blockade. However, these therapeutic opportunities remain largely supported by preclinical and early translational evidence and require validation in prospective clinical studies [40,41,42].

### 6.3. Secondary Bile Acids and Immune Suppression

Primary bile acids synthesized by the liver undergo extensive microbial transformation within the intestine, generating secondary bile acids such as deoxycholic acid (DCA) and lithocholic acid (LCA). Although these metabolites are important regulators of host metabolism, excessive accumulation may exert pro-inflammatory and pro-carcinogenic effects [35,42].

Experimental studies have shown that secondary bile acids can influence macrophage polarization, cytokine secretion, epithelial barrier function, and immune-cell recruitment. In CRC, dysregulated bile acid metabolism has been associated with chronic inflammation, oxidative stress, DNA damage, and tumor progression [12,42].

Recent evidence further suggests that bile acids may modulate antitumor immunity by influencing dendritic-cell function, T-cell activation, and immune checkpoint signaling. Consequently, alterations in microbiota-driven bile acid metabolism may contribute to the establishment of an immunosuppressive tumor microenvironment that favors immune escape and limits responsiveness to checkpoint blockade [12,35].

### 6.4. Succinate: A Potential Mediator of Immunotherapy Resistance

Among emerging microbial metabolites, succinate has recently attracted considerable attention for its potential roles in immune regulation and immunotherapy resistance. Elevated succinate concentrations have been observed in inflammatory and neoplastic conditions and may influence both immune and epithelial cell function [13,36].

Recent studies suggest that succinate derived from microbes may impair antitumor immunity by modulating macrophage activity, inflammatory signaling, and interferon-related pathways. Of particular interest, recent mechanistic evidence has linked *F. nucleatum*-associated succinate production to suppression of the cGAS–STING–IFN-β signaling axis, a pathway critically involved in dendritic cell activation and antitumor immune priming [13].

Because cGAS–STING signaling is increasingly recognized as a key determinant of immune checkpoint inhibitor responsiveness, disruption of this pathway by microbial metabolites could represent a previously underappreciated mechanism of immunotherapy resistance in MSS CRC. Collectively, these findings identify succinate as an emerging mediator of microbiome-associated immune regulation and a promising candidate linking gut dysbiosis to immune escape. However, the current evidence is derived predominantly from mechanistic and preclinical studies, and further experimental, translational, and prospective clinical investigations are required to validate its role as both a biomarker and a potential therapeutic target in MSS CRC.

### 6.5. Inosine and Purine Metabolism

Inosine is a purine metabolite produced by specific intestinal bacteria and has emerged as one of the best-characterized microbiota-derived metabolites capable of enhancing antitumor immunity under appropriate biological conditions [36,37].

The immunological effects of inosine appear to depend on the activation state of immune cells and the cytokine milieu. In the presence of appropriate co-stimulatory signals, inosine may support Th1 polarization and promote interferon gamma (IFN-γ) production, thereby enhancing antitumor immune responses [36].

These findings are particularly important because they demonstrate that microbiota-derived metabolites should not be viewed as uniformly immunosuppressive. Instead, microbial metabolites exert diverse, context-dependent immunological effects, with inosine as a notable example that can enhance antitumor immunity and improve responsiveness to immune checkpoint blockade under appropriate biological conditions. Consequently, manipulation of microbial purine metabolism may represent a promising strategy for microbiome-guided immune re-sensitization in MSS CRC.

### 6.6. Microbial Metabolites as Functional Biomarkers and Therapeutic Targets

Collectively, current evidence suggests that microbial metabolites may represent more robust predictors of immunotherapy responsiveness than microbial taxonomy alone. While bacterial composition varies considerably between individuals and populations, many microbial communities converge toward similar metabolic outputs. Therefore, functional metabolomic profiling may provide a more accurate representation of microbiome–host interactions [35,36,37,38].

This concept is particularly relevant for MSS CRC, where multiple bacterial species may contribute to immune resistance through shared metabolic pathways. Metabolites such as butyrate, indole derivatives, bile acids, succinate, and inosine serve as functional mediators that influence immune surveillance, antigen presentation, cytokine signaling, and responsiveness to checkpoint inhibitors.

Accordingly, future precision immuno-oncology approaches may increasingly integrate microbiome-derived metabolomics with metagenomics, transcriptomics, and tumor immune profiling to identify patients most likely to benefit from microbiome-targeted interventions.

An important consideration is that the immunological effects of microbial metabolites are highly context-dependent and should not be interpreted as uniformly beneficial or detrimental. Their biological activity is influenced by metabolite concentration, microbial community composition, tumor molecular characteristics, host immune status, and interactions with other microbial and host-derived factors. Consequently, the same metabolite may exert distinct, or even opposing, immunological effects depending on the biological context. This complexity underscores the importance of functional metabolomic profiling when evaluating metabolite-based biomarkers and microbiome-targeted therapeutic strategies in MSS colorectal cancer.

To facilitate the understanding of the complex interactions between gut microbiome dysbiosis, microbial metabolism, tumor immune regulation, and immunotherapy responsiveness in MSS colorectal cancer, Figure 2 provides an integrated overview of the microbiome–metabolite–immune axis. The schematic compares the microbial composition of a healthy colon with MSS CRC-associated dysbiosis, highlighting representative bacterial taxa and metabolite profiles that collectively shape the tumor immune microenvironment, influence responsiveness to ICIs, and provide potential targets for microbiome-based therapeutic interventions.

## 7. Microbiome-Based Therapeutic Strategies to Overcome Immunotherapy Resistance in MSS Colorectal Cancer

Growing evidence indicates that the gut microbiome is not only a biomarker of immunotherapy responsiveness but also a potentially modifiable therapeutic target. Unlike tumor genetics, which are relatively stable and difficult to manipulate, the intestinal microbiota can be altered through dietary interventions, probiotics, postbiotics, fecal microbiota transplantation (FMT), selective microbial depletion, and engineered bacterial therapies. Consequently, microbiome-targeted interventions have emerged as promising investigational strategies with the potential to overcome immune resistance and enhance the efficacy of ICIs in MSS CRC [35,36,37,38].

The rationale for microbiome modulation is supported by accumulating evidence that microbial communities influence dendritic cell activation, antigen presentation, T-cell priming, cytokine production, immune checkpoint signaling, and microbial metabolite profiles. Therefore, therapeutic manipulation of the microbiome may represent a feasible strategy for restoring antitumor immunity and converting immunologically cold tumors into immune-responsive disease [36,37,38].

### 7.1. Fecal Microbiota Transplantation

FMT is currently the most comprehensive microbiome-targeted intervention for modifying the intestinal microbial ecosystem. By transferring an entire microbial community from a healthy donor or an immunotherapy responder, FMT has the potential to restore microbial diversity, re-establish beneficial metabolic pathways, and modulate both mucosal and systemic immune responses [43,44]. Unlike probiotics or postbiotics, which primarily influence selected microbial taxa or metabolic pathways, FMT aims to reconstitute the entire intestinal ecosystem, thereby simultaneously affecting microbial composition, metabolite production, epithelial barrier integrity, and host immune regulation.

Interest in FMT as an adjunct to immunotherapy originated from landmark clinical studies in metastatic melanoma, where transplantation of microbiota from patients with durable responses to ICI successfully restored responsiveness in a subset of previously anti-PD-1-refractory patients. These studies provided the first clinical evidence that the composition of the gut microbiome can influence the efficacy of immune checkpoint blockade [44]. Subsequent immune profiling demonstrated successful donor microbiota engraftment, increased intratumoral CD8^+^ T-cell infiltration, enhanced antigen presentation, activation of interferon signaling pathways, and reduced frequencies of immunosuppressive myeloid cell populations. Although comparable clinical experience in CRC remains limited, preclinical studies suggest that FMT may enhance antitumor immunity by increasing beneficial commensal bacteria, restoring short-chain fatty acid production, enhancing dendritic cell maturation, and promoting CD8+ T-cell activation. Additionally, FMT may reduce the abundance of pathobionts associated with immune suppression, including *F. nucleatum* and other CRC-associated bacterial species [34,43]. Recent translational studies further support the concept that microbiome remodeling may improve immune responsiveness when combined with ICIs or multimodal therapeutic strategies, although definitive clinical evidence in MSS CRC is still lacking [45,46,47].

The most recent evidence synthesis further supports the clinical potential of FMT as an adjunct to immunotherapy. A 2025 meta-analysis including 10 prospective studies involving 164 patients with advanced or refractory solid tumors reported a pooled objective response rate (ORR) of 43% (95% CI, 35–51%) following FMT combined with immune checkpoint inhibitors. Subgroup analyses demonstrated significantly higher response rates among patients receiving combined anti-PD-1 plus anti-CTLA-4 therapy (ORR 60%; 95% CI, 45–73%) compared with anti-PD-1 monotherapy (ORR 37%; 95% CI, 27–49%; *p* = 0.01). The pooled complete and partial response rates were 12% and 34%, respectively. Importantly, treatment-related adverse events were generally manageable, with grade 1–2 events occurring in 42% of patients and grade 3–4 events in 37%, supporting the feasibility of microbiome modulation as an adjunct to cancer immunotherapy [45]. Despite these encouraging findings, FMT remains an investigational strategy. Major challenges include donor selection and screening, variability in microbial engraftment, differences in FMT preparation and administration protocols, long-term safety considerations, and the lack of standardized biomarkers to predict treatment response [46,48]. Consequently, larger randomized multicenter clinical trials integrating longitudinal microbiome profiling, immune monitoring, metabolomics, and biomarker-guided patient stratification are required before FMT can be incorporated into routine immunotherapy strategies for MSS CRC.

The principal clinical studies evaluating FMT in combination with ICIs, along with their major clinical and immunological findings, are summarized in Table 2.

### 7.2. Probiotics and Next-Generation Beneficial Bacteria

Growing evidence suggests that specific microbial taxa may influence responsiveness to immune checkpoint blockade across multiple malignancies, supporting the rationale for probiotic-based immune modulation strategies [50,51].

Traditional probiotics have been investigated for their ability to improve gut microbial balance and reduce inflammation. However, recent attention has shifted toward next-generation probiotics with specific immunomodulatory properties, including *Akkermansia muciniphila*, *Bifidobacterium* spp., *Faecalibacterium prausnitzii*, and selected members of the Lachnospiraceae family [36,38].

Among these organisms, *A. muciniphila* has attracted particular interest due to its association with improved responses to immune checkpoint blockade across several cancers. Experimental studies indicate that *A. muciniphila* can enhance antigen presentation, stimulate dendritic-cell maturation, strengthen epithelial barrier integrity, and promote CD8+ T-cell activation [36,52].

Similarly, *Bifidobacterium* species have been shown to improve antitumor immunity by enhancing dendritic cell function and increasing interferon signaling. Because these microorganisms contribute to the production of beneficial metabolites, including SCFAs and inosine, they may simultaneously improve the metabolic and immunological components of the tumor microenvironment [36,37].

Although encouraging, current evidence remains largely preclinical, and the efficacy of probiotic supplementation in MSS CRC patients undergoing immunotherapy requires further validation in controlled clinical trials.

### 7.3. Postbiotics and Metabolite-Based Therapeutics

An emerging alternative to microbiome transplantation involves the direct administration of microbial metabolites or microbial-derived bioactive molecules, often referred to as postbiotics. This strategy aims to bypass the complexity and variability of microbial engraftment while preserving beneficial immunological effects [35,36,37,38].

Several metabolites discussed in the previous section have already demonstrated immunomodulatory potential. Butyrate may enhance epithelial barrier integrity and support CD8+ T-cell activity, whereas inosine can promote Th1 polarization and IFN-γ production. Conversely, therapeutic reduction in immunosuppressive metabolites such as succinate may help restore effective antitumor immunity [36,37,38].

Compared with FMT, postbiotic approaches offer greater standardization, easier regulatory approval, and improved reproducibility. Consequently, metabolite-based therapies may be among the most promising avenues for future microbiome-guided immunotherapy.

### 7.4. Selective Microbial Depletion and Precision Microbiome Editing

Because certain bacterial taxa contribute to immune suppression and therapy resistance, selective elimination of pathogenic microorganisms represents another potential therapeutic strategy. Unlike broad-spectrum antibiotics, which may disrupt beneficial microbial communities, precision microbiome editing aims to selectively target harmful bacteria while preserving beneficial taxa [13,36].

Potential approaches include narrow-spectrum antibiotics, bacteriophage therapy, CRISPR-based microbial editing, antimicrobial peptides, and small molecules that selectively inhibit bacterial virulence factors. *Fusobacterium nucleatum* is currently the most attractive target for such interventions due to its established role in immune modulation and CRC progression [13,23].

However, microbial ecosystems are highly interconnected, and eliminating a single microorganism may have unintended ecological consequences. Therefore, future precision microbiome editing strategies will likely require personalized microbiome profiling and systems-level approaches.

### 7.5. Engineered Bacterial Therapeutics

Advances in synthetic biology have enabled the development of genetically engineered microorganisms capable of delivering therapeutic molecules directly to the tumor microenvironment. These live bacterial therapeutics represent one of the most innovative directions in microbiome-based oncology [53].

Engineered bacteria can be designed to produce cytokines, immune stimulatory molecules, checkpoint-modulating agents, tumor antigens, or metabolite-modifying enzymes. Because bacteria naturally colonize hypoxic and necrotic tumor regions, they may serve as highly efficient therapeutic delivery systems [53].

Although most engineered bacterial platforms remain in preclinical development, their ability to combine targeted delivery with immune activation makes them particularly attractive for overcoming the profound immune resistance observed in MSS CRC.

### 7.6. Combination Strategies with Immune Checkpoint Inhibitors

Given the multifactorial nature of immune resistance in MSS CRC, microbiome-targeted interventions will likely be most effective when integrated into combination treatment strategies. Potential approaches include combining microbiome modulation with PD-1/PD-L1 inhibitors, CTLA-4 blockade, chemotherapy, radiotherapy, anti-angiogenic agents, TGF-β inhibitors, cancer vaccines, and adoptive cell therapies [6,36].

In this context, the microbiome should not be viewed as an isolated therapeutic target but rather as a modulatory platform that can enhance the efficacy of existing anticancer therapies. Future clinical trials will need to determine which microbiome interventions, patient populations, and treatment combinations produce the greatest therapeutic benefit.

### 7.7. Current Challenges and Future Perspectives

Despite remarkable progress, several obstacles continue to limit the clinical implementation of microbiome-based therapies. These include interindividual variability in microbiome composition, differences in diet and lifestyle, methodological heterogeneity across studies, lack of standardized biomarkers, and uncertainty regarding the durability of microbiome modulation [36,37,38].

Furthermore, it remains unclear whether microbial taxonomy, microbial metabolites, immune signatures, or integrated multi-omics profiles will ultimately provide the most clinically useful predictors of immunotherapy response. Addressing these challenges will be essential for translating microbiome-based interventions into routine oncological practice.

Collectively, current evidence suggests that microbiome-targeted therapies could be an important component of future immunotherapy strategies for MSS CRC. However, their successful implementation will require a precision medicine framework integrating microbiome profiling, immune characterization, metabolomics, and clinical stratification. Current evidence remains limited by methodological heterogeneity, differences in microbiome assessment techniques, variability in dietary and environmental exposures, and the lack of standardized predictive biomarkers, all of which complicate translation into clinical practice [48,50,51].

While microbiome-based interventions represent promising therapeutic tools, their successful clinical implementation requires accurate identification of patients most likely to benefit from such strategies. Consequently, recent research has increasingly focused on integrating metagenomics, metabolomics, transcriptomics, spatial profiling, and artificial intelligence to develop predictive models of immunotherapy responsiveness.

A broad range of microbiome-targeted therapeutic approaches is currently being investigated to overcome immune resistance and improve immunotherapy responsiveness in MSS CRC. The principal strategies, their mechanisms of action, and their current stage of development are summarized in Table 3.

## 8. Multi-Omics and Precision Immuno-Microbiome Oncology in MSS Colorectal Cancer

The growing recognition of the gut microbiome as a determinant of antitumor immunity has generated considerable interest in developing precision-medicine approaches to predict immunotherapy responsiveness. However, the clinical translation of microbiome research faces a major challenge: substantial interindividual variability in microbial composition, metabolite production, immune status, dietary habits, and tumor biology. Consequently, single biomarkers are unlikely to adequately capture the complexity of microbiome–host interactions in colorectal cancer [37,54,55].

To address this limitation, recent research has increasingly focused on integrating multiple layers of biological information, including metagenomics, metatranscriptomics, metabolomics, transcriptomics, spatial immune profiling, and artificial intelligence (AI)-based predictive modeling. Collectively, these approaches form the foundation of precision immuno-microbiome oncology, an emerging field aimed at identifying clinically actionable microbiome–immune signatures to predict treatment response and guide personalized therapeutic strategies [37,54,55].

### 8.1. Metagenomics: Beyond Microbial Taxonomy

Metagenomic sequencing has transformed microbiome research by enabling comprehensive characterization of microbial communities without the need for conventional culture methods. In CRC, metagenomic analyses have consistently demonstrated enrichment of microbial signatures associated with carcinogenesis, including *F. nucleatum*, enterotoxigenic *Bacteroides fragilis*, pks-positive *E. coli*, *P. micra*, and other pathobionts [24,25].

While taxonomic profiling has significantly improved our understanding of CRC-associated dysbiosis, its predictive value for immunotherapy remains limited. Different microbial communities may produce similar metabolic outputs, whereas identical bacterial species may exhibit substantial functional variability depending on strain composition and ecological context [55]. Consequently, metagenomics alone may be insufficient to accurately predict immunotherapy responsiveness in MSS CRC.

Nevertheless, microbial composition remains an important first-level biomarker and serves as the foundation for more advanced multi-omics integration strategies.

### 8.2. Metatranscriptomics and Functional Microbial Activity

Unlike metagenomics, which identifies the presence of microbes, metatranscriptomics evaluates microbial gene expression and therefore provides information about biological activity. This distinction is particularly important because bacterial abundance does not necessarily reflect microbial function [34].

Recent studies suggest that transcriptionally active microbial communities may be more strongly associated with tumor biology than taxonomic composition alone. For example, biofilm-associated bacteria may exhibit enhanced expression of virulence factors, inflammatory mediators, and metabolic pathways that influence immune cell recruitment and tumor progression [34].

From a clinical perspective, metatranscriptomic profiling may help distinguish passive microbial colonization from biologically relevant microbial activity. Consequently, future predictive models of immunotherapy response may benefit from integrating both microbial composition and microbial transcriptional activity.

### 8.3. Metabolomics: Functional Readouts of Microbiome–Host Interactions

Among all microbiome-related technologies, metabolomics may provide the most direct assessment of microbiome function. Rather than focusing on bacterial identity, metabolomic analyses quantify the bioactive molecules produced by microbial communities and their interactions with host metabolic pathways [35,36,37,38].

As discussed in the previous section, metabolites such as butyrate, inosine, indole derivatives, succinate, and secondary bile acids directly influence immune-cell differentiation, cytokine production, antigen presentation, and immune checkpoint responsiveness. Therefore, metabolomic signatures may represent more robust biomarkers of therapeutic response than bacterial taxonomy alone [35,36,37,38].

Several investigators have proposed that future patient stratification strategies should incorporate microbial metabolite profiling alongside conventional molecular biomarkers. Such approaches may facilitate identification of patients with favorable immunometabolic profiles who are more likely to benefit from microbiome-targeted interventions and immune checkpoint blockade.

### 8.4. Tumor Transcriptomics and Immune Gene Signatures

Microbiome-derived signals ultimately exert their effects through host cellular pathways. Consequently, integration of tumor transcriptomic data with microbiome profiling provides a powerful approach for understanding host–microbe interactions.

Transcriptomic analyses can identify immune activation signatures, interferon-related pathways, cytokine networks, T-cell exhaustion markers, antigen-presentation machinery, and immune checkpoint expression profiles. In MSS CRC, such analyses may reveal whether dysbiosis-associated microbial communities are linked to immune exclusion, myeloid-driven immunosuppression, or altered interferon signaling [8,9].

Recent studies suggest that combining microbial signatures with immune gene expression profiles improves predictive performance compared with either modality alone. This supports the concept that microbiome data should be interpreted within the broader context of the tumor immune microenvironment.

### 8.5. Spatial Transcriptomics and the Geography of Tumor–Microbiome Interactions

One of the most exciting recent developments is the application of spatial transcriptomics to CRC research. Unlike conventional sequencing methods, spatial transcriptomics preserves tissue architecture and allows simultaneous analysis of gene expression within specific anatomical locations [56,57].

This technology is particularly relevant in CRC because microbial communities are not uniformly distributed throughout the tumor. Instead, microorganisms often localize within biofilms, invasive margins, hypoxic regions, or specific stromal niches. Spatial transcriptomics, therefore, provides an unprecedented opportunity to investigate how microbial localization influences immune-cell recruitment, macrophage polarization, T-cell exclusion, and therapeutic responsiveness [56,57].

Future studies integrating microbial mapping with spatial immune profiling may reveal previously unrecognized mechanisms of immune resistance in MSS CRC.

### 8.6. Artificial Intelligence and Machine Learning-Based Predictive Models

The enormous complexity of microbiome–host interactions presents significant analytical challenges. Consequently, AI and machine learning approaches have become increasingly important tools for identifying predictive microbiome signatures.

Recent machine learning models have successfully integrated microbiome composition, clinical variables, immune parameters, and multi-omics datasets to predict CRC prognosis and immunotherapy responsiveness [54]. These algorithms may identify nonlinear relationships that cannot be detected through conventional statistical approaches.

Importantly, AI-based models may facilitate personalized treatment selection by identifying patient-specific microbial and immune profiles associated with favorable therapeutic outcomes. As datasets continue to expand, machine learning is expected to become an essential component of precision microbiome oncology.

Despite their considerable promise, current AI- and machine learning-based models face several important limitations that may hinder their clinical implementation. Many predictive algorithms have been developed using relatively small, single-center cohorts and heterogeneous datasets that differ in sequencing technologies, bioinformatic pipelines, sample processing, and patient characteristics. These sources of variability may reduce model generalizability, limit external validation, and compromise reproducibility across independent populations. Consequently, future progress will require standardized data acquisition, multicenter prospective studies, transparent model development, rigorous external validation, and independent clinical testing before AI-driven microbiome biomarkers can be reliably implemented in routine precision oncology.

### 8.7. Toward Precision Immuno-Microbiome Oncology

The ultimate goal of multi-omics integration is to develop clinically actionable precision medicine frameworks. In such a model, patient stratification would no longer rely solely on MSI status, tumor mutational burden, or PD-L1 expression. Instead, therapeutic decision-making would incorporate microbial composition, microbial activity, metabolite profiles, immune signatures, and computational prediction models [37,54,55].

For MSS CRC, this approach may be particularly valuable because resistance to immunotherapy is multifactorial and cannot be explained by a single biomarker. Multi-dimensional profiling may therefore identify subsets of MSS patients who could benefit from microbiome-targeted interventions combined with immune checkpoint blockade.

Collectively, these advances suggest that the future of CRC immunotherapy may lie not only in targeting tumors but also in understanding and therapeutically manipulating the complex ecosystem that surrounds them.

## 9. Proposed Integrated Model of Microbiome-Associated Immune Resistance and Therapeutic Re-Sensitization in MSS Colorectal Cancer

The emerging evidence discussed throughout this review supports a paradigm shift in our understanding of immunotherapy resistance in MSS CRC. Traditionally, resistance to immune checkpoint inhibitors has been attributed primarily to tumor-intrinsic factors, including low tumor mutational burden, limited neoantigen generation, defective antigen presentation, and an immunologically “cold” tumor microenvironment. However, growing data indicate that these mechanisms alone cannot fully explain the heterogeneous responses observed among patients with MSS CRC [6,8].

We propose an integrated model in which gut microbiome dysbiosis acts as an upstream regulator of immune resistance through multiple interconnected biological pathways. In this framework, dysbiosis is characterized by enrichment of pathobionts such as *F. nucleatum*, enterotoxigenic *B. fragilis*, pks-positive *E. coli*, *P. micra*, and other CRC-associated microorganisms, together with depletion of beneficial metabolite-producing commensals [13,24,25]. These microbial alterations contribute to epithelial barrier dysfunction, chronic inflammation, activation of oncogenic signaling pathways, and remodeling of the tumor microenvironment.

The immunological consequences of dysbiosis are mediated through both direct and indirect mechanisms. Direct microbial–host interactions promote immune exclusion, suppression of dendritic-cell function, expansion of myeloid-derived suppressor cells, polarization of tumor-associated macrophages toward immunosuppressive phenotypes, and impairment of cytotoxic T-cell and natural killer cell activity [13,19,20,21]. Simultaneously, microbial metabolites—including short-chain fatty acids, indole derivatives, bile acids, succinate, and inosine—modulate immune-cell differentiation, cytokine signaling, interferon responses, and checkpoint inhibitor sensitivity [12,13,35,36,37,38,39,40,41,42].

Collectively, these processes converge to establish the characteristic immune-resistant phenotype of MSS CRC. Rather than functioning as isolated mechanisms, immune exclusion, myeloid-driven immunosuppression, T-cell dysfunction, metabolic reprogramming, and microbial signaling appear to operate as components of a coordinated dysbiosis-driven network. This model may explain why many MSS tumors remain resistant to checkpoint blockade despite expression of targetable immune pathways.

Importantly, the same framework also identifies multiple opportunities for therapeutic intervention. Strategies such as fecal microbiota transplantation, next-generation probiotics, postbiotic administration, selective microbial depletion, engineered bacterial therapeutics, and metabolite-based interventions may partially reverse dysbiosis-associated immune suppression and restore antitumor immunity [34,37,43,44,48,50,51,52,53,54,55]. In this context, microbiome-targeted therapies should not be viewed as alternatives to immunotherapy but rather as complementary approaches that can enhance responsiveness to existing ICIs.

To integrate the diverse mechanisms discussed throughout this review, Figure 3 presents a comprehensive conceptual model illustrating how tumor-intrinsic molecular alterations, tumor metabolic reprogramming, gut microbiome dysbiosis, and immune remodeling converge to establish the immune-resistant phenotype of MSS CRC. The schematic also highlights how microbiome-based therapeutic interventions may modulate these interconnected pathways, restore antitumor immunity, and improve responsiveness to ICI.

## 10. Future Directions and Clinical Perspectives

Despite remarkable progress in understanding the relationship between the gut microbiome and antitumor immunity, several important questions remain unresolved. Current evidence strongly supports the biological relevance of microbiome-mediated immune regulation in colorectal cancer; however, translation into routine clinical practice remains limited by methodological, biological, and regulatory challenges [36,37].

One of the most important priorities for future research is identifying reliable biomarkers to predict immunotherapy responsiveness. Current approaches often focus on individual bacterial taxa, yet accumulating evidence suggests that microbial metabolites, functional microbial activity, and integrated multi-omics signatures may provide more robust and reproducible biomarkers than taxonomy alone [35,36,37,38]. Their successful clinical implementation will require standardized methodologies for sample collection, sequencing, bioinformatic analysis, and multicenter validation. AI and machine-learning approaches are expected to facilitate the integration of these high-dimensional datasets and improve biomarker discovery and patient stratification [50,51,54].

Future studies should increasingly shift from descriptive association studies toward mechanistic and interventional investigations. Emerging technologies, including spatial transcriptomics, spatial metabolomics, and microbial localization analyses, may provide unprecedented insights into tumor–microbiome interactions and the mechanisms underlying immune exclusion and resistance to ICI in MSS CRC [56].

Collectively, these advances support the development of microbiome-guided precision immuno-oncology. By integrating microbial profiling with immune characterization, spatial biology, metabolomics, and computational modeling, future precision strategies may enable more accurate patient stratification and expand the clinical benefits of immunotherapy to the large population of patients with MSS CRC who currently derive limited benefit from immune checkpoint blockade.

## 11. Conclusions

Current evidence indicates that the gut microbiome represents an important modulator of antitumor immunity and therapeutic responsiveness in MSS CRC. Rather than acting in isolation, microbiome dysbiosis interacts with tumor-intrinsic molecular alterations, metabolic reprogramming, stromal remodeling, and immune regulatory pathways to reinforce the immune-resistant phenotype characteristic of MSS tumors. These advances support a paradigm shift from descriptive microbiome profiling to mechanistic, biomarker-driven precision immuno-oncology. Future progress will depend on integrating microbiome characterization with immune profiling, metabolomics, spatial biology, and computational approaches to identify patients most likely to benefit from microbiome-targeted interventions. Although microbiome-based therapeutic strategies—including fecal microbiota transplantation, next-generation probiotics, postbiotics, engineered bacterial therapeutics, and metabolite-based interventions—remain largely investigational, they represent promising adjunctive approaches for overcoming immune resistance. Continued mechanistic, translational, and clinical research will be essential to establish standardized, evidence-based strategies that expand the clinical benefits of ICI for the large population of patients with MSS CRC.

## Figures and Tables

**Table 1:** Major gut microbial taxa associated with immune modulation and potential resistance to immune checkpoint inhibitors in microsatellite-stable colorectal cancer (MSS CRC).

Microorganism	Major Virulence Factors/ Mechanisms	Effects on the Tumor Microenvironment	Potential Impact on Immunotherapy	Current Level of Evidence	Representative References
***Fusobacterium*** ***nucleatum***	FadA, Fap2, TIGIT interaction, NF-κB activation	T-cell suppression, NK-cell inhibition, MDSC/TAM recruitment, chronic inflammation	Immune evasion, impaired antitumor immunity, and potential resistance to immune checkpoint inhibitors	Mechanistic, translational, early clinical	[13,14,15,16,17]
**Enterotoxigenic** ***Bacteroides fragilis***	BFT (fragilysin), IL-17/STAT3 activation	Chronic inflammation, Th17 polarization, epithelial barrier disruption	Pro-tumor immune remodeling and immune suppression	Mechanistic, observational	[18,19,20]
**pks-positive** ***Escherichia coli***	Colibactin production, DNA damage	DNA damage, genomic instability, inflammation, epithelial stress, mutational signatures	Tumor evolution, immune escape, and potential therapeutic resistance	Mechanistic, observational	[21,22,23,24]
***Parvimonas micra***	Metabolite-mediated signaling; inflammatory activation	Pro-inflammatory tumor microenvironment, immune modulation	CRC progression and potential contribution to immune resistance	Observational, translational	[25,26]
***Peptostreptococcus anaerobius***	PI3K/Akt activation, ROS generation	Tumor promotion, inflammatory signaling, immune modulation	Potential contributor to immune suppression and therapeutic resistance	Mechanistic, observational	[27,28]
**Biofilm-associated communities**	Spatial microbial organization, polymicrobial interactions	Chronic inflammation, epithelial barrier disruption, Treg/M2 macrophage enrichment	Maintenance of immune-suppressive niches and reduced immunotherapy responsiveness	Mechanistic	[29,30]

**Table 2:** Representative clinical studies investigating fecal microbiota transplantation (FMT) in combination with immune checkpoint inhibitors (ICIs).

Study	Cancer Type	Study Design	Main Clinical Findings	Main Immunological Findings
Baruch et al., 2021 [49]	Metastatic melanoma	Phase I	Objective responses and durable disease stabilization in a subset of anti-PD-1 refractory patients	Donor microbiota engraftment, increased CD8^+^ T-cell infiltration, enhanced IFN signaling
Davar et al., 2021 [44]	Metastatic melanoma	Phase I	Restoration of anti-PD-1 responsiveness in selected patients	Increased antigen presentation, activation of cytotoxic T cells, reduction in immunosuppressive myeloid cells
Current CRC clinical studies (2023–2025)	MSS/MSI-H CRC	Early-phase clinical studies	Feasibility demonstrated; efficacy remains under investigation	Preliminary immune remodeling after microbiome modulation
Lin et al., 2025 (Meta- analysis) [45]	Advanced solid tumors	Systematic review and meta-analysis (10 studies, *n* = 164)	Pooled ORR 43%; higher ORR with anti-PD-1 + anti-CTLA-4 (60%) vs. anti-PD-1 alone (37%); acceptable safety profile	Supports the clinical feasibility of microbiome-mediated enhancement of antitumor immunity

Summary of representative clinical studies evaluating FMT combined with immune checkpoint inhibitors. Although most available clinical evidence comes from metastatic melanoma, recent studies and ongoing clinical trials suggest that microbiome modulation may be a promising strategy to improve immunotherapy responsiveness in CRC.

**Table 3:** Emerging microbiome-based therapeutic strategies for overcoming immune resistance in microsatellite-stable colorectal cancer (MSS CRC), including their current level of evidence and principal limitations.

Strategy	Mechanism of Action	Immune Effects	Current Level of Evidence	Key Limitations
Fecal microbiota transplantation (FMT)	Restoration of microbial diversity	Enhanced antigen presentation and T-cell activation	Early clinical studies	Donor selection and screening; variability in engraftment; lack of standardized protocols; limited MSS CRC-specific clinical evidence
Next-generation probiotics	Enrichment of beneficial taxa	Improved immune regulation and metabolite production	Preclinical/ Early clinical	Optimal strain selection; colonization efficiency; limited clinical validation
Postbiotics	Administration of microbial metabolites	Direct modulation of immune pathways	Preclinical	Optimal dosing and formulation; pharmacokinetic uncertainty; lack of clinical studies
Selective microbial depletion	Removal of pathobionts (e.g., *F. nucleatum*)	Reduced immune suppression	Preclinical	Off-target microbiome disruption; antimicrobial resistance; durability of response
Bacteriophage therapy	Targeted elimination of pathogenic bacteria	Precision microbiome editing	Experimental	Narrow host specificity; bacterial resistance; manufacturing and regulatory challenges
Engineered bacterial therapeutics	Local delivery of immune-stimulatory molecules	Tumor-targeted immune activation	Preclinical	Biosafety concerns; regulatory approval; genetic stability; limited human data
Metabolite-based interventions	Restoration of beneficial metabolite profiles	Immune re-sensitization	Emerging	Incomplete mechanistic understanding; biomarker selection; lack of prospective clinical validation
Combination with ICIs	Microbiome modulation plus checkpoint blockade	Enhanced antitumor immunity	Ongoing investigation	Limited prospective randomized trials; optimal patient selection; treatment standardization

## Data Availability

The data used to support the findings of this study are available from the corresponding author upon reasonable request.
